# Integra's legacy unveiled: expert panel recommendations summarizing 25 years of experience in head and neck reconstruction

**DOI:** 10.1016/j.jpra.2025.02.021

**Published:** 2025-02-27

**Authors:** Franco Bassetto, Juan Carlos Lopez-Gutierrez, Riccardo Giunta, Benedetta Scucchi, Mark Singh, Cesare Tiengo

**Affiliations:** a Plastic and Reconstructive Surgery Unit, Department of Neurosciences, University of Padua, Padua, Italy; b Pediatric Surgery Department, La Paz University Hospital, Madrid, Spain; c Division of Hand, Plastic and Aesthetic Surgery, University Hospital LMU, 81377 Munich, Germany; d Oral and Maxillofacial Surgery Department, Bristol Royal Infirmary, Bristol, UK

**Keywords:** dermal regeneration template, head, neck, reconstruction, surgery

## Abstract

**Background:**

Integra® Dermal Regeneration Template (IDRT) is used for its ability to facilitate wound closure and mimic various skin functions, marking a breakthrough in medical technology.

**Methods:**

Through a synthesis of expert opinions, clinical experiences, and published evidence, this review sheds light on current applications of IDRT in head and neck wound care.

**Results:**

This review covers the application of IDRT for post-oncological surgery reconstructions, trauma-induced injuries, and scar corrections. It focuses on the use of IDRT in specific anatomical regions (scalp, temporal, orbital, nasal areas, neck, and ears) and in pediatric and older patients. The economic aspects, including cost-effectiveness and value in the healthcare system, are also examined.

**Conclusions:**

The insights presented aim to inform clinicians and encourage the broader integration of IDRT into clinical practice, enhancing patient outcomes in wound management.

## Introduction

Dermal skin substitutes have emerged as a major innovation in wound care.^1^ These are scaffolds that, after placement, undergo degradation and are subsequently replaced by extracellular matrix produced by fibroblasts in the host tissue.^2^ Among different dermal substitutes, Integra® Dermal Regeneration Template (IDRT; Integra LifeSciences, Princeton, NJ, USA) holds the distinction of being the pioneering product.^3^ Conceived by Yannas and Burke in the 1980s, IDRT was born from the need for surgeons to have an accessible dermal regeneration technique.^3^, ^4^, ^5^

The unique composition of IDRT, a bioengineered 3D matrix built on a collagen–glycosaminoglycan matrix, offers immediate wound coverage. Associated with a split-thickness skin graft (STSG), it fosters the regeneration of functional dermal tissue.^6^ Notably, IDRT addresses some of the longstanding challenges in repairing skin damage, including contractures and scars. Thus, it represents a favorable alternative to conventional skin grafting and flap reconstruction. Although they can provide immediate coverage, these methods are hindered by limitations in supply, difficulties in covering exposed structures, and complications at the donor site.^6^^,^^7^

IDRT has rapidly gained importance in reconstructive surgery, becoming the preferred choice for treating several conditions, ranging from burns and ulcers to traumatic injuries and excision lesions. The clinical outcomes, characterized by sustained engraftment paired with superior cosmetic and functional results, have been extensively documented.^7^, ^8^, ^9^, ^10^ Even with the advent of advanced techniques and procedures, the relevance of IDRT remains undiminished.^11^

In June 2023, a panel of international plastic surgeons with profound expertise in the application of IDRT gathered in Madrid to thoroughly examine the use of IDRT in head and neck anatomical regions. The meeting was organized by Polistudium, a scientific consultancy company, and was funded by Integra. The discussion, enriched by clinical insights, covered a spectrum of cases spanning post-oncological surgery reconstructions to trauma-induced injuries and scars from burns or prior surgeries. Supplemented by a critical review of the available evidence, this article presents the insights from this meeting, with a specific focus on the application of IDRT in the scalp, temporal region, orbital region, nasal region, neck, and ears.

## Integra in scalp reconstruction

Beyond its biological function of protecting the skull and brain, the scalp, particularly the hair, often symbolizes vitality, youth, and health.^12^ Consequently, any disfigurement of the scalp can have profound psychological implications.^13^

The scalp is composed of discrete zones characterized by varying degrees of tissue tightness and laxity.^14^ Surgeons must consider this structural heterogeneity when selecting the optimal approach to scalp reconstruction.^14^ Furthermore, the surgical strategy must consider the potential ramifications on essential aesthetic attributes, such as hairline, to minimize the deleterious effects of scarring, alopecia, and disfigurement.^14^ Remarkably, antecedent scarring or fibrosis can reduce the efficacy of reconstruction, particularly when loco-regional flap techniques are used.^15^ Traditional reconstruction methods, especially in patients who have undergone multiple surgeries or radiation, can result in scarring.^16^ Given the scalp's proximity to the facial region, any distortion can magnify the perceived anomaly.^17^

The application of IDRT in scalp reconstruction has garnered substantial attention, especially in addressing diverse etiologies, and it is supported by the most robust evidence involving head and neck regions, as suggested by recent comprehensive reviews on the topic.^7^^,^^18^^,^^19^

A notable area of focus is the role of IDRT in post-oncological surgical reconstruction. IDRT has demonstrated commendable efficacy, especially in patients who have received surgical interventions or adjuvant radiotherapy.^18^^,^^19^ Such patients often present with prior incisions, scarring, and radiation-induced damage that might reduce the success of conventional reconstructive procedures. Further strengthening the case for IDRT is its suitability for patients diagnosed with recurrent and aggressive tumors who are submitted to rigorous tumor surveillance. Indeed, IDRT facilitates unobstructed access to the tumor site, thus ensuring accurate follow-up evaluations.^7^^,^^18^

IDRT is also useful in reconstruction after injury, especially in challenging situations. However, its effectiveness is contingent upon tailored application according to the specific requirements of each case.^19^

## Integra in temporal region reconstruction

The temporal region plays a subtle yet important role in the overall harmony of facial aesthetics. Disruptions or abnormalities in this area can alter facial symmetry, leading to potential psychological implications.^20^

The delicate nature of the area, combined with its proximity to essential structures, requires precision. Traditional reconstructive techniques can lead to scarring or asymmetry.^21^

The use of IDRT in reconstructing the temporal region is still an emerging field of study. Halmy et al. reported the successful application of IDRT for managing a large (180 cm^2^), recurrent skin tumor of the temporal area.^21^ A subsequent skin grafting procedure was performed on the 28th day after the placement of IDRT. Both IDRT and skin grafting exhibited a 100% success rate, signifying the potential of IDRT in ensuring both functional and aesthetic outcomes following surgical interventions for skin tumors on the skull.

Bocchiotti et al. described the case of a 69-year-old man who underwent resection of a sizable, bloody, exophytic, and ulcerated lesion in the right temporoparotideal area, compounded by several actinic keratoses.^22^ Given the patient's underlying comorbidities and the specific attributes of the surgical defect, a sternocleidomastoideus (SCM) flap was used for the reconstruction process to minimize the risk of skin paddle necrosis. To further enhance the reconstruction, especially considering the depth of the surgical site and the potential for postsurgical scar retraction, the muscle was shielded with single-layer IDRT. This was complemented by a skin graft from the patient's left thigh. One year after surgery, the patient had achieved both the oncological and reconstructive objectives. Drawing from this experience, combining an SCM flap with IDRT and a skin graft might represent a viable strategy for reconstructing the temporal region, particularly when other reconstructive options are unsuitable.

## Integra in orbital region reconstruction

The eyes are central to human expression, communication, and interpersonal connection.^23^ A harmonious and symmetrical orbital area not only ensures optimal visual function but also heavily influences appearance, self-esteem, and how others perceive an individual.^24^

Given the intricate anatomy, including the eyelids, lacrimal system, and bony orbit, ensuring functional integrity and preserving aesthetic appeal when reconstructing the orbital region is paramount.^25^ Traditional reconstructive methods might lead to complications such as scarring, which can be highly distressing.^26^

The use of IDRT in orbital region reconstruction is supported by multiple studies. Thinda et al. documented the application of IDRT to reconstruct traumatic tissue loss in the periocular area.^26^ This was evidenced by a case involving a 36-year-old woman who presented with a devastating tissue defect ranging from the medial canthus to the temporal fossa and from the pretarsal skin to the brow after a motor vehicle accident. Given the severity of the tissue loss, conventional methods, such as autografts or allografts, were deemed unsuitable. The use of IDRT resulted in a successful reconstructive outcome.

Patel et al. further bolstered the versatility of IDRT by illustrating its use in the case of a 63-year-old man affected by an invasive basal cell carcinoma with multiple right eyelid masses.^27^ After orbital exenteration (OE), IDRT was used to cover the surgical defect, which was integrated and vascularized within 3 weeks. The robustness of this graft allowed for further surgical intervention when the carcinoma recurred. At a follow-up of 47 weeks, the patient showed a full recovery without any complications.

Ameloot et al. described the case of a 42-year-old man diagnosed with a moderately differentiated epidermoid carcinoma infiltrating the orbit, necessitating total exenteration.^28^ The patient's rehabilitation involved the insertion of IDRT followed by a subsequent thin skin graft. Ozgonul et al. reported the use of IDRT in 5 cases, with successful outcomes and sockets becoming prosthesis-ready within 8 weeks in all patients.^29^

Chen et al. highlighted the application of IDRT for traumatic periocular tissue loss in four patients unsuitable for primary closure or immediate flap reconstruction.^30^ Notably, three of the four patients exhibited full or remarkable healing with IDRT, reducing the need for additional reconstructive procedures.^30^ The authors pointed out that IDRT should be considered a primary treatment option in patients who might experience lagophthalmos or deformity from primary closure of the periocular defect and for whom immediate flap reconstruction is not deemed the best choice. This is especially recommended for younger patients who may lack easily movable surrounding tissue.^30^

The use of IDRT in reconstruction following OE, alone or with regional flaps, was also supported by a study by Monjanel et al.^31^ This approach also reduced surgical morbidity and hospital stay. Importantly, pre- and postoperative radiotherapy did not appear to delay socket healing.^31^

Finally, a recent case by Bothwick et al. emphasized the applicability of IDRT in complex situations.^32^ In this case, a 68-year-old patient with multiple medical conditions underwent OE, radiotherapy, and subsequent reconstruction. Even in an irradiated area devoid of periosteum, IDRT was effectively integrated, underscoring the benefits of less invasive reconstructive techniques, especially in patients with multiple comorbidities and a noticeable risk of cancer recurrence.^32^

Overall, current evidence supports the role of IDRT in orbital reconstruction, showcasing its versatility and efficacy in various clinical scenarios. Research in this field is ongoing and will further elucidate the capabilities and applications of IDRT in this setting.

**Figure 1:**
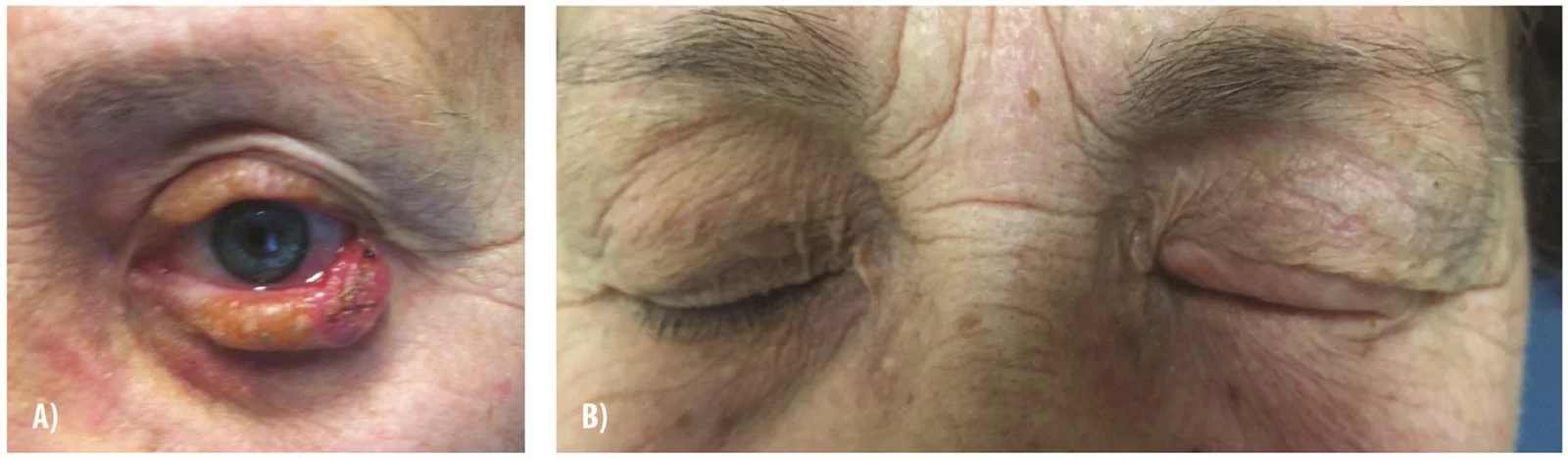
**Eyelid reconstruction with Integra Dermal Regeneration Template after basal cell carcinoma excision. (A)** An 85-year-old woman with a basal cell carcinoma on the left lower eyelid. Following resection, Integra Dermal Regeneration Template was applied to address the defect. **(B)** Outcome at 12 months post-reconstruction reveals well-integrated skin grafts and restoration of eyelid anatomy, preserving both function and aesthetics.

We also present a case of eyelid reconstruction involving basal cell carcinoma resection (Figure 1). Following excision of the carcinoma and application of IDRT, the patient demonstrated excellent functional and aesthetic recovery at the 12-month follow-up, with well-integrated grafts and restored eyelid anatomy.

## Integra in nasal and lip region reconstruction

Beyond its aesthetic significance, the nose also performs vital functions related to respiration and olfaction.^33^ Given its prominence and functionality, any deformity or alteration of the nasal area can have profound physiological and psychological consequences.^34^^,^^35^

The anatomy of the nose, encompassing cartilage, bone, and soft tissues, requires precise restoration to ensure preservation of both form and function.^36^ Traditional reconstructive techniques can cause complications such as scarring or undesirable changes in nasal shape.^37^

Despite the relatively limited evident, the current literature on the application of IDRT in nasal reconstruction documents cases of rhinoplasty, nasal reconstruction with exposed cartilage, nasal tip reconstruction, nasal dorsum reconstruction or augmentation, as well as reconstruction of open nasal wounds, complex pediatric nasal lesions, and nasal floor.^37^, ^38^, ^39^, ^40^, ^41^, ^42^, ^43^, ^44^, ^45^, ^46^, ^47^

The longest cohort study was a retrospective analysis of the application of IDRT for nasal reconstruction on 35 patients presenting 36 nasal defects.^37^ These defects covered 76 aesthetic subunits, providing a diverse range of cases for analysis. Of these, 58 nasal and 8 extra-nasal aesthetic subunits benefited from IDRT reconstruction, while 10 subunits were reconstructed using a flap technique.^37^ The outcomes were largely positive: 29/36 defects healed uneventfully, accounting for an 80.5% complication-free healing rate.^37^ The 100% complication-free healing of all reconstructed nasal tips/columella and nasal dorsum was noteworthy, with a mean defect size of 9.6±1.6 cm^2^. However, retraction of the alar rim was seen in 33.3% (4/12) of the patients with nasal alar involvement. Additionally, the formation of a fistula occurred in the nasal sidewall in 4.8% (1/21) of cases.

**Figure 2:**
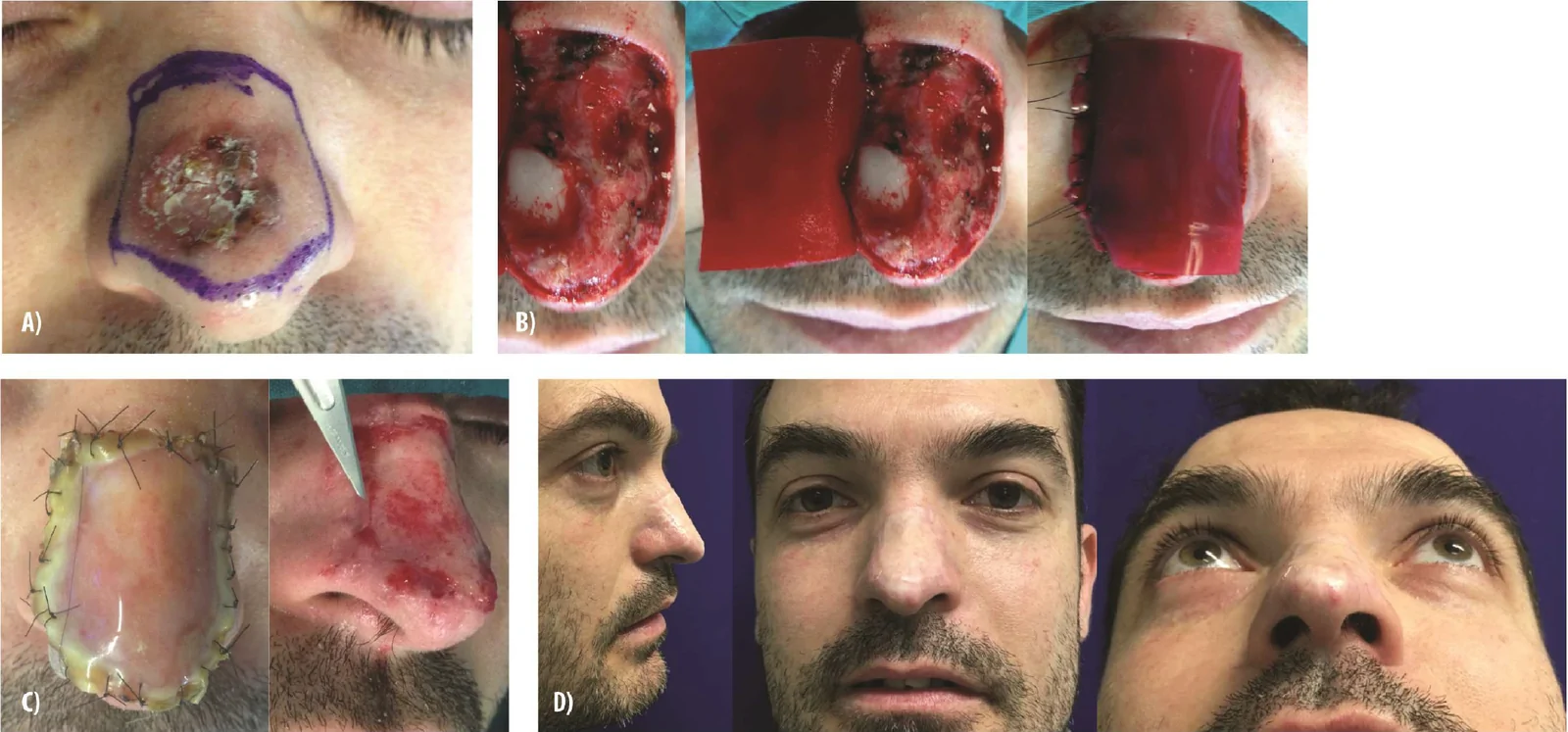
**Reconstruction of the external nose with Integra Dermal Regeneration Template. (A)** A 37-year-old man presenting with a basal cell carcinoma on the external nose. **(B)** Following oncologic resection, a defect exposing the right alar cartilage is evident. **(C)** Placement of Integra Dermal Regeneration Template over the defect, followed by a split-thickness skin graft 3 weeks later after dermal maturation. **(D)** The final outcome after 8 months demonstrates successful reconstruction with restored contour and no significant scarring.

**Figure 3:**
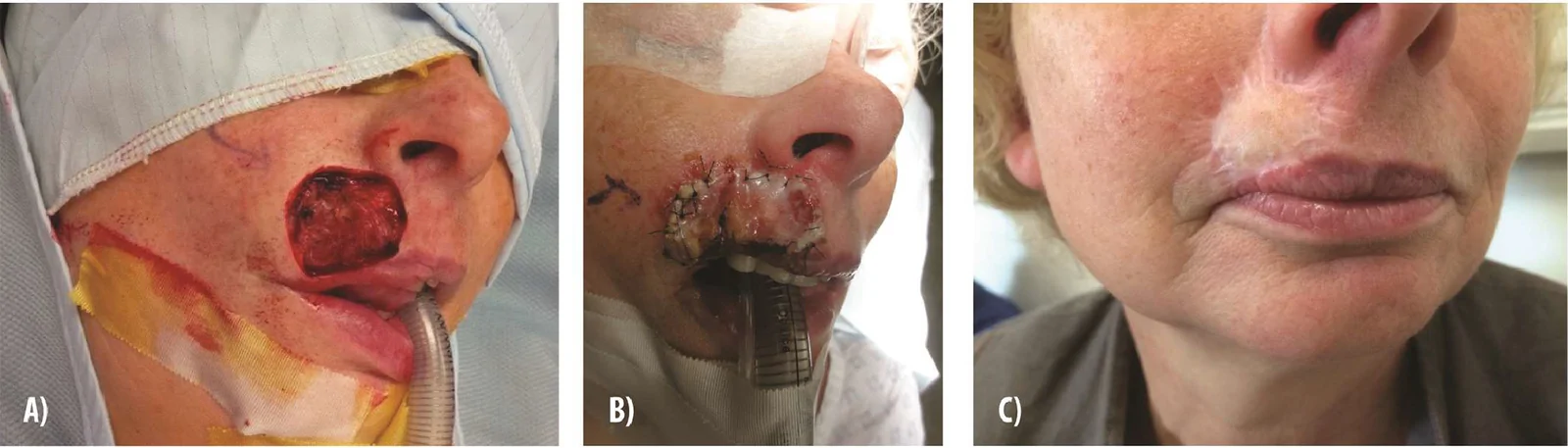
**Upper lip reconstruction involving the vermillion border using Integra Dermal Regeneration Template. (A)** Defect of the upper lip, including the vermillion border, following basal cell carcinoma excision. **(B)** Two weeks post-Integra Dermal Regeneration Template application, demonstrating dermal regeneration and preparation for grafting. **(C)** Final appearance post-split-thickness skin graft placement, showing no lip retraction or distortion of the vermillion border. Aesthetic and functional integrity is preserved.

We include here a representative case of nasal reconstruction (Figure 2) highlighting the successful use of IDRT in restoring form and function. The patient exhibited excellent integration of the dermal template and minimal scarring. Furthermore, although there is no established evidence on the use of IDRT for lip reconstruction, we provide a case involving the vermillion border (Figure 3) for educational purposes. This case demonstrates the preservation of both aesthetic and functional integrity in a complex upper lip defect following basal cell carcinoma resection.

The application of IDRT in nasal and lip region reconstruction showcases promising outcomes. Most defects healed without complications, highlighting the potential of this method in nasal reconstruction. However, the presence of region-specific complications underscores the importance of tailoring reconstruction techniques.

## Integra in neck reconstruction

The neck, while often overlooked in favor of facial features, holds great importance in contributing to an individual's overall appearance and sense of identity.^20^ The skin in this area is distinctively thin and flexible, making it prone to scarring or complications following surgical procedures. Additionally, the neck houses vital structures, making surgeries more intricate.^48^ Various studies have investigated the efficacy of IDRT in diverse neck reconstruction scenarios.

Burd and Wong first introduced 1-stage IDRT reconstruction in head and neck defects, showcasing its potential for scarless repair, particularly in basal cell carcinoma cases.^44^ This study revealed reduced operating time, no delay for frozen sections, and acceptable long-term aesthetic results. The work of Felcht et al. expanded on this by exploring a 2-stage reconstruction strategy using IDRT.^49^ This approach was a viable alternative for reconstructing extended head and neck defects, offering good cosmetic results and stable scars, especially in older patients.

The use of IDRT in neck burns has been explored in multiple articles. Hoinoiu et al. showed the effectiveness of IDRT in treating post-burn hypertrophic scars and contractures of the neck and lower face.^50^ Patients expressed satisfaction with the outcomes, and the positive impact of good skin elasticity alleviated any minor issues.

Furthermore, Gaviria and Gómez-Ortega introduced a 1-stage reconstruction approach for a neck burn using a single-layer IDRT combined with STSG.^51^ Their study, involving 9 patients, showed the effectiveness, safety, and associated excellent outcomes of IDRT.

Mujahid et al. delved into the outcome of successful IDRT graft take and STSG after the release of post-burn neck contracture.^52^ The analysis of 70 cases indicated a high success rate in terms of complete revascularization and skin graft take, showcasing IDRT and STSG as promising modalities in burn management and reconstructive surgery. In addition, Hunt et al. presented their initial experience of using IDRT to treat post-burn anterior cervical neck contracture.^53^ Their study included 5 patients who underwent IDRT grafting over 12 months. Despite achieving a superior cosmetic result in neck contracture and hypertrophic scarring, more than 50% of contracture recurrences occurred, suggesting ineffective immobilization of the graft and overgranulation as factors contributing to contracture recurrence.

The abovementioned studies collectively underscore the versatility of IDRT in neck reconstruction, demonstrating its potential in various scenarios, from tumors to burns. The diverse 1- and 2-stage approaches and the utilization of collagen–glycosaminoglycan matrices offer surgeons effective tools to achieve satisfactory outcomes in aesthetics, functionality, and patient satisfaction. The evolving role of IDRT in neck reconstruction will likely see further refinement and expansion with ongoing research and clinical applications.

**Figure 4:**
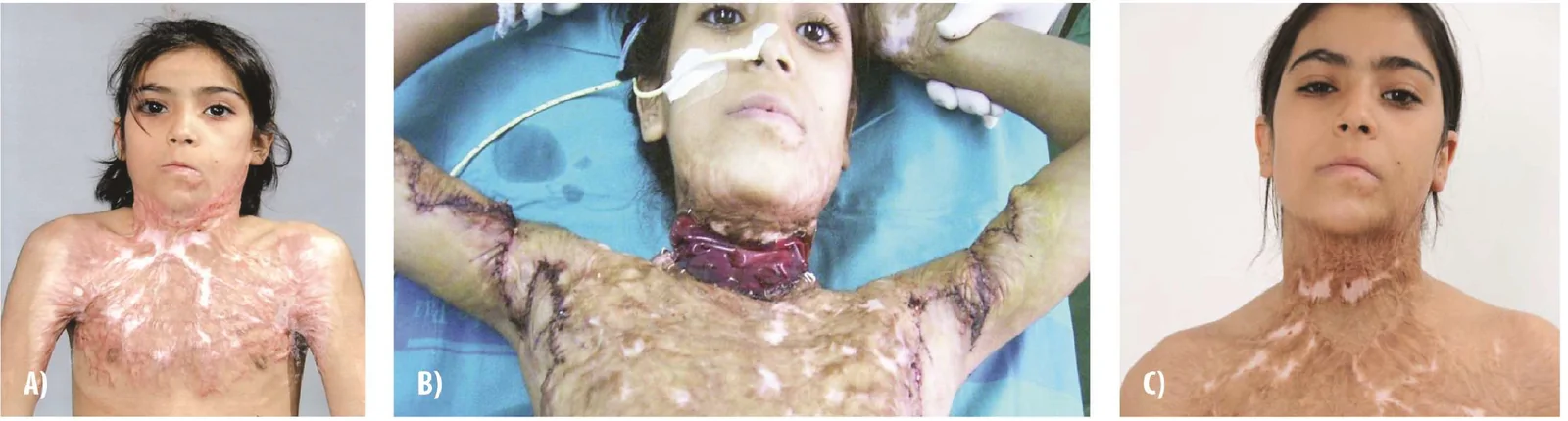
**Reconstruction of post-burn neck and axillary contractures with Integra Dermal Regeneration Template. (A)** Severe post-burn neck and axillary contractures in a patient injured during the Iraq War, significantly limiting range of motion. **(B)** Surgical release of contractures, with bilateral Z-plasty in the axilla and application of Integra Dermal Regeneration Templateto the neck defects. **(C)** Outcome at 2 years post-reconstruction shows substantial improvement in neck mobility and cosmetic appearance, with no contracture recurrence.

In support of these findings, we provide a case involving severe post-burn neck and axillary contractures (Figure 4). Following surgical release with bilateral Z-plasty and IDRT application to the neck defects, the patient showed substantial improvement in mobility and cosmetic appearance. No recurrence of contractures was observed at the 2-year follow-up, underscoring the efficacy of this approach.

## Integra in ear reconstruction

More than just a hearing organ, the ear contributes greatly to the overall aesthetics of the facial profile.^20^ Given its complex cartilaginous structure and detailed anatomy, achieving a natural appearance of the ear after reconstruction can be complicated.^54^ Overcoming scarring and ensuring proper hearing are crucial.^55^ Additionally, the ear-exposed nature makes it susceptible to external factors, thus requiring the utmost precision in surgical interventions. IDRT has emerged as an innovative tool for ear reconstruction.

A 2012 case report by Reiffel et al. showed the potential of IDRT in treating recurrent auricular keloids.^56^ They reported the case of a 23-year-old woman with recurrent auricular keloid after various treatments, including surgical excision, corticosteroid injection, and radiation. The patient's keloid was re-excised, and the resultant exposed auricular perichondrium was immediately covered with IDRT. The patient subsequently received high-dose-rate brachytherapy (1500 cGy) on the first and second postoperative days, followed by a definitive STSG placement 3 weeks after the initial surgery. The patient's recovery was smooth with no complications. Remarkably, 27 months after the STSG placement, there was no sign of keloid formation in either the auricular recipient or the thigh donor sites.

In 2018, Daya et al. described the use of IDRT in a patient who had sustained a traumatic right ear soft tissue avulsion.^57^ On examination, there was evident skin avulsion exposing the cartilage from the concha, scapha, and antihelix of the right ear. The chosen treatment sequence commenced with debridement, followed by the application of an IDRT graft. The dressing was then changed every 3 days for 3 weeks. Once adequate vascularization was evident, an STSG was placed. This approach underscored the capability to reconstruct a significant skin avulsion from the right ear.^57^ Moreover, it ensured minimal donor-site morbidity and eliminated the need for local rotational flaps, thus preserving the possibility for future reconstructions.^57^

Additionally, a study by Lei et al. lends further support to the efficacy of IDRT in this setting.^41^ One-stage reconstruction using IDRT demonstrated a mean time of healing of 23.4 days postoperatively, with no hyperpigmentation, tumor recurrence, or retraction noted. Patients were followed up for 25.7 months, and high acceptance and satisfaction with the outcome were observed in all cases. The authors concluded that single-stage reconstruction is not only aesthetically acceptable but also more time and cost-effective than traditional 2-stage techniques.^41^

**Figure 5:**
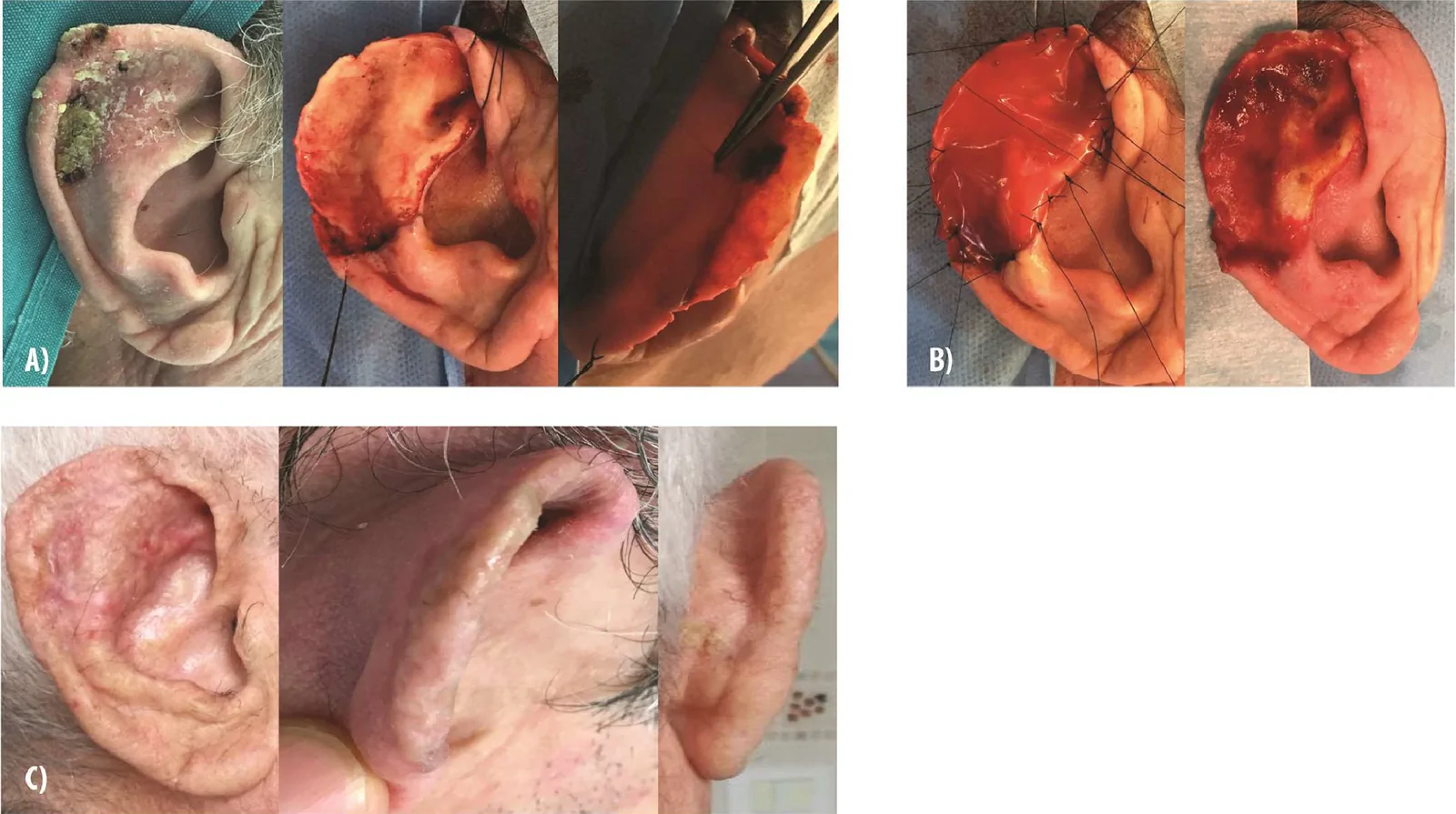
**Reconstruction of the external ear with Integra Dermal Regeneration Template following oncologic resection. (A)** Squamous cell carcinoma of the external right ear with cartilage exposure after radical cancer resection. **(B)** Immediate application of Integra Dermal Regeneration Template graft, with the dermal regeneration surface ready for grafting after 3 weeks. **(C)** Outcome at 1-year follow-up showing stable and anatomical ear reconstruction.

Collectively, these case studies highlight the adaptability and effectiveness of IDRT in ear reconstruction, affirming its potential as a suitable choice in such challenging cases. We also present a case of external ear reconstruction following radical resection of squamous cell carcinoma (Figure 5). This case illustrates immediate IDRT application over exposed cartilage, with successful dermal regeneration observed after 3 weeks and stable, anatomical ear reconstruction achieved at the 1-year follow-up.

## Use of Integra in special populations

### Pediatric patients

The management of complex pediatric surgical wounds poses a considerable challenge for reconstructive surgeons. Children's unique physiology and any congenital conditions contribute to a distinctive paradigm for wound closure in this population.^58^ Furthermore, as children grow, their skin grows as well. Children are notably susceptible to dog bites in the head and face regions.^59^ A meticulous reconstructive strategy is essential, considering the fragile state of an infant, the need to avoid reinterventions, the active pediatric lifestyle, and the shared goal of minimizing donor-site morbidity.^58^

The application of IDRT has shown promising results in reconstructing various congenital and acquired conditions in children.^60^ Such conditions encompass congenital melanocytic nevi and absent cranium, burn contractures, aplasia cutis, myelomeningoceles, vascular malformations, and complex nasal lesions.^46^^,^^58^^,^^61^, ^62^, ^63^, ^64^

An important advantage of using IDRT lies in its capacity to reduce the need for traditional large, full-thickness skin grafts. Instead, STSGs can be employed. This shift in procedure markedly reduces the associated donor-site morbidity and is associated with favorable outcomes for total scalp and extensive burn scar reconstruction in children.^65^, ^66^, ^67^

IDRT can also be considered in nasal reconstruction. It is common practice to employ full-thickness skin grafts in nasal reconstruction because of their enhanced color-matching properties and reduced secondary contraction compared with STSGs.^68^^,^^69^ Nevertheless, this approach is often associated with increased skin recoil and pronounced scar visibility due to the lack of natural wrinkles in the pediatric skin.^46^

While IDRT offers a promising alternative to pediatric reconstructive surgery, dedicated research on relatively large populations is necessary.

### Older patients

The use of IDRT in older patients represents a promising solution to the challenges posed by reconstructive efforts in this population.^70^ A predominant concern is the increased vulnerability of seniors to skin cancers and traumatic injuries, and IDRT indeed offers a recommended treatment modality.^11^^,^^71^ Notably, post-wide excision reconstruction becomes particularly challenging within the senior cohort.^7^^,^^72^ Although options such as free tissue transfers, local flaps, and skin grafts exist, they have notable disadvantages, especially in older patients presenting with advanced local cancers, systemic diseases, or a historical recurrence of scalp skin cancers. In the latter, there is an inherent risk because using a flap could potentially bury a recurrent tumor.

Corradino et al. highlighted a method involving an initial surgical procedure for tumor removal and subsequent positioning of IDRT, followed by a second procedure for definitive reconstruction.^72^ This 2-stage process yielded promising outcomes, with full graft take observed across all cases and favorable cosmetic and functional results documented. Additionally, early tumor recurrence was detected in 2 cases, underscoring the utility of this approach in early detection.

Mogedas-Vegara et al*.* embarked on a retrospective analysis of 70 patients, mostly males with comorbidities, with the aim of ascertaining the success rates of IDRT and STSG.^70^ Their analysis revealed an impressive success rate for both IDRT and STSG. Furthermore, the postoperative complication rate was relatively low, and no significant associations were found between the variables and the primary and secondary endpoints.

Echoing these findings, Bernstein et al. conducted a retrospective analysis on patients aged >80 years who underwent full-thickness scalp reconstruction post-tumor excision.^73^ Their results substantiated the efficacy of the 2-stage approach, with most patients obtaining a 100% graft take. This study further cemented the 2-stage approach with IDRT as a viable reconstructive strategy, especially for managing full-thickness scalp defects after oncological resection in older patients.

Overall, IDRT is a commendable solution for older patients requiring reconstructive surgeries, particularly tumor-related excisions. The 2-stage approach, proven by multiple studies, offers both clinical efficacy and improved patient outcomes.

## Cost and health economics considerations

The perception of IDRT as a relatively costly option versus autografts is a primary factor limiting its adoption in clinical practice. Schiavon et al. comprehensively compared the costs associated with IDRT-based treatments versus flap surgeries.^74^ Their focus was primarily on patients who experienced scalp defects with exposed bone. Demographic and clinical factors were statistically consistent between the 2 groups. Encouragingly, all patients successfully healed, displaying durable and high-quality closure. Economically, the median total cost for the IDRT group and the flap surgery group was similar (€11,121 [IQR: 8,327–15,571] vs€7,259 [IQR: 1,852–24,443];p = 0.34). A subset analysis was conducted on patients showing defects >100 cm^2^. Interestingly, in this scenario, the treatment with IDRT appeared less costly at €11,825 (IQR: 10,695–15,751) than the flap group at €23,244 (IQR: 17,348–26,942).^74^

Although diverse healthcare systems may present different economic scenarios and their conclusions cannot be universally extrapolated, according to the above findings, IDRT could be economically viable.^74^^,^^75^ However, a holistic understanding of the economic implications also warrants a brief overview of the broader social impact. The potential reduction in the number of hospitalizations using IDRT not only translates into medical cost savings but also minimizes the loss of income for patients. Furthermore, there is a ripple effect with the reduced demand on healthcare professionals' time and improved quality of life for patients. In light of these multifaceted benefits, IDRT offers both medical and socioeconomic advantages that cannot be ignored.

## Expert opinion and future perspectives

Over the past few decades, the landscape of skin reconstruction has transformed considerably, influenced not only by technological advancements but also by evolving patient perspectives.^76^ Since entering the market in 1981, IDRT has swiftly become one of the most favored dermal substitutes, especially for the intricate regions of the head and neck.^77^ Over the years, the changing tide of patient perceptions has ushered in a heightened focus on minimizing scarring, optimizing aesthetic outcomes, and understanding the social relevance of reconstructive dermal choices.^78^ The role of IDRT is paramount in this regard. Given its primary aim of creating functional neodermis and aesthetic enhancement, the long-term results from its application have been overwhelmingly positive.^79^ The product closely mimics the patient's natural skin, thus easily concealing the treated area. This ability is invaluable, particularly after procedures such as skin cancer excision and traumatic injuries, for which preservation of original anatomy, volume, and dimension is pivotal.^80^

The commendable advantages of IDRT span its capacity to maintain space, promote vascularization, reduce contraction and fibroblastic infiltration, and ensure stable reconstruction.^8^ Particularly noteworthy is its efficacy in addressing cartilage exposure post-skin cancer removal, where outcomes with IDRT are optimized through meticulous debridement and ensuring a vascular-rich wound bed.^9^ This is very useful in complex regions such as the nose, where maintaining the inherent contour and function is essential. The same principles could be applied to the oral cavity, where regenerating the mucosa and safeguarding the orbicularis muscle become critical.

Furthermore, IDRT synergizes well with microsurgical procedures, delivering remarkable results in thin tissue regions such as the earlobe and mucosa. Given the vast experience available, IDRT can integrate seamlessly into the routine practice of many plastic surgeons, particularly those well-versed in microsurgery and cosmetic procedures. The benefits of IDRT can be particularly relevant in challenging populations, such as patients with underlying health conditions, older individuals, and children, for whom minimally invasive approaches are favored. Moreover, its importance must be considered in emergency scenarios in which conventional surgical routes are impractical or additional scarring is undesirable.

However, IDRT is not always the best approach. It is positioned as a preferred option in the reconstructive ladder, typically recommended before flaps and post-skin grafts. However, it is crucial to juxtapose it with other techniques, weighing the pros and cons to ensure the best outcome. Its benefits shine particularly bright in specific demographics.

The versatility of IDRT offers room for innovative integrations, such as combining it with negative pressure in cases of bone involvement, which translates into a life-saving indication, or pairing it with tissue expanders and hair transplants to enhance scalp coverage and cosmetic results. There is also a potential avenue to explore its synergistic use in post-radiotherapy scenarios.

A concerted educational push is imperative to maximize the benefits and appropriate utilization of IDRT, especially in head and neck region reconstruction. Only with a profound understanding of its capabilities, limitations, and potential can we ensure the best outcomes for patients.

## Conflicts of interest

Assistance for the preparation of this manuscript was funded by Integra. The meeting which led to the preparation of this manuscript was funded by Integra. The authors declare that they have no further conflict of interest.

## References

[1] Alrubaiy L., KK Al-Rubaiy (2009). Skin substitutes: a brief review of types and clinical applications. Oman Med J.

[2] Rehim S.A., Singhal M., Chung KC. (2014). Dermal skin substitutes for upper limb reconstruction: current status, indications, and contraindications. Hand Clin.

[3] Yannas I.V., Burke JF. (1980). Design of an artificial skin. I. Basic design principles. J Biomed Mater Res.

[4] Yannas I.V., Burke J.F., Gordon P.L., Huang C., Rubenstein RH. (1980). Design of an artificial skin. II. Control of chemical composition. J Biomed Mater Res.

[5] Dagalakis N., Flink J., Stasikelis P., Burke J.F. (1980). Yannas IV. Design of an artificial skin. Part III. Control of pore structure. J Biomed Mater Res.

[6] Taupin P., Gandhi A., Saini S. (2023). Integra® dermal regeneration template: From design to clinical use. Cureus.

[7] Magnoni C., De Santis G., Fraccalvieri M., Bellini P., Portincasa A., Giacomelli L. (2019). Integra in scalp reconstruction after tumor excision: recommendations from a multidisciplinary advisory board. J Craniofac Surg.

[8] Chang D.K., Louis M.R., Gimenez A., Reece E.M. (2019). The basics of integra dermal regeneration template and its expanding clinical applications. Semin Plast Surg.

[9] Chalmers R.L., Smock E., Geh JLC. (2010). Experience of Integra(®) in cancer reconstructive surgery. J Plast Reconstr Aesthet Surg.

[10] Papa G., Spazzapan L., Pangos M., Delpin A., Arnez ZM. (2014). Compared to coverage by STSG grafts only reconstruction by the dermal substitute Integra® plus STSG increases TcPO2 values in diabetic feet at 3 and 6 months after reconstruction*. G Chir.

[11] Scalise A., Torresetti M., Di Benedetto G. (2020). Reconstruction of full-thickness soft tissue defects with integra: risk factors and treatment algorithm. Plast Reconstr Surg Glob Open.

[12] Lane C., Lin A., Goyal N. (2023). Scalp and calvarium reconstruction. Otolaryngol Clin North Am.

[13] Sarwer D.B., Siminoff L.A., Gardiner H.M., Spitzer JC. (2022). The psychosocial burden of visible disfigurement following traumatic injury. Front Psychol.

[14] Seery GE. (2001). Scalp surgery: anatomic and biomechanical considerations. Dermatologic Surg.

[15] Ferreira R.A., Contin L.A., Rocha V.B., Calil JA. (2022). Reconstruction of scalp anterior hairline with tissue expansion and skin flap. Skin Appendage Disord.

[16] Park H., Min J., Oh T.S., Jeong W.S., Choi J-W (2022). Scalp reconstruction strategy based on the etiology of the scalp defects. J Craniofac Surg.

[17] Bhattacharya V. (2012). Management of soft tissue wounds of the face. Indian J Plast Surg.

[18] Depani M., Grush A.E., Parham M.J., Jones L.M., Thornton JF. (2022). Use of biologic agents in nasal and scalp reconstruction. Semin Plast Surg.

[19] Watts V., Attie M.D., McClure S. (2019). Reconstruction of complex full-thickness scalp defects after dog-bite injuries using dermal regeneration template (Integra): case report and literature review. J Oral Maxillofac Surg.

[20] Jones H.E., Faulkner H.R., Losken A. (2022). The psychological impact of aesthetic surgery: a mini-review. Aesthetic Surg journal Open Forum.

[21] Halmy C., Nádai Z., Csőre K., Vajda A., Tamás R. (2013). [The use of Integra dermal regeneration matrix in the surgical treatment of a recurrent, extensive, malignant skin lesion in the temporal region]. Orv Hetil.

[22] Bocchiotti M.A., Raimondo L., Germano S., Ruka E., Frenello A., Garzaro M., Pecorari G. (2017). Use of the sternocleidomastoid flap in association with a dermal regeneration template and a skin graft in the temporal region reconstruction. Innov Surg Sci.

[23] Naik MN. (2016). Periocular Aesthetics: An Emerging Era. J Cutan Aesthet Surg.

[24] Tower R.N., Soparkar C.N.S., Patrinely JR. (2007). Perspectives on periocular asymmetry. Semin Plast Surg.

[25] Anlatici R., Ozerdem O.R. (2016). Reconstruction of eyelids and related structures. J Craniofac Surg.

[26] Thinda S., Wright H.V., Mawn LA. (2012). Integra bilayer matrix wound dressing closure of large periorbital traumatic wound. Arch Ophthalmol.

[27] Patel S.Y., Tamboli D.A., Mancini R. (2018). Two-stage rapid exenteration reconstruction to allow early radiation therapy for an aggressive orbital cancer. Int Ophthalmol.

[28] Ameloot F., Mezzine H., Khairallah G., Hayek G., Zaidi M., Lhuillier L., Talbi M., Sot M., Perone JM. (2019). Reconstruction of exenteration socket with Integra® dermal substitute and skin graft. J Fr Ophtalmol.

[29] Ozgonul C., Diniz Grisolia A.B., Demirci H. (2018). The use of Integra® dermal regeneration template for the orbital exenteration socket: a novel technique. Ophthal Plast Reconstr Surg.

[30] Chen T.A., Ayala-Haedo J.A., Blessing N.W., Topping K., Alabiad C.R., Erickson BP. (2018). Bioengineered dermal substitutes for the management of traumatic periocular tissue loss. Orbit.

[31] Monjanel B., Baillif S., Lagier J., Gastaud L., Poissonnet G., Martel A. (2021). Efficacy and safety of an artificial dermal graft for the reconstruction of exenterated sockets: a preliminary report. Graefe's Arch Clin Exp Ophthalmol = Albr von Graefes Arch fur Klin und Exp Ophthalmol.

[32] Bothwick V., Polanik M., Lujan-Hernandez J., Akyurek M. (2023). Orbital exenteration reconstruction for medically complex patients: bilaminate dermal substitute as an alternative to major surgery. J Craniofac Surg.

[33] Freeman SC, Karp DA, Kahwaji CI. Physiology, Nasal. In Treasure Island (FL); 2023. 30252342

[34] Choi KY. (2015). Analysis of facial asymmetry. Arch Craniofacial Surg.

[35] Rifkin W.J., Kantar R.S., Ali-Khan S., Plana N.M., Diaz-Siso J.R., Tsakiris M. (2018). Facial disfigurement and identity: a review of the literature and implications for facial transplantation. AMA J Ethics.

[36] Fischer H., Gubisch W. (2008). Nasal reconstruction: a challenge for plastic surgery. Dtsch Arztebl Int.

[37] Moratin K., Koch P-S, Benecke J., Orouji A., Bauer C., Faulhaber J. (2019). Reconstruction of nasal defects with dermal skin substitutes-a retrospective study of 36 defects. J Cutan Med Surg.

[38] Seth A.K., Ratanshi I., Dayan J.H., Disa J.J., Mehrara BJ. (2019). Nasal reconstruction using the integra dermal regeneration template. Plast Reconstr Surg.

[39] Mumtaz S., Patel H., Singh M. (2020). Use of Integra® dermal regeneration template and flowable matrix to reconstruct an oral cavity defect involving the nasal floor. Br J Oral Maxillofac Surg.

[40] Planas J. (2011). The use of Integra^TM^ in rhinoplasty. Aesthet Plast Surg.

[41] Lei Y-H, Huang S-H. (2022). Single-stage acellular dermal matrix reconstruction of defects in the nose and ears with exposed cartilage: a prospective case series. BMC Surg.

[42] Applebaum M.A., Daggett J.D., Carter WL. (2015). Nasal tip reconstruction using Integra bilayer wound matrix: an alternative to the forehead flap. Eplasty.

[43] Duteille F., Tilliet Le Dentu H., Atlan M., Perrot P. (2019). Use of Integra flowable wound matrix for nasal dorsum reconstruction or augmentation: A series of 6 cases. J Plast Reconstr Aesthet Surg.

[44] Burd A., Wong PSY. (2010). One-stage Integra reconstruction in head and neck defects. J Plast Reconstr Aesthet Surg.

[45] Blanco N.M., Edwards J., Zamboni WA. (2004). Dermal substitute (Integra) for open nasal wounds. Plast Reconstr Surg.

[46] Grunwaldt L.J., Adetayo O.A., MacIsaac Z.M., Losee J.E., Kumar AR. (2014). Successful reconstruction of complex pediatric nasal lesions: improving outcomes using dermal regenerative templates. Plast Reconstr surgery Glob open.

[47] Koenen W., Felcht M., Vockenroth K., Sassmann G., Goerdt S., Faulhaber J. (2011). One-stage reconstruction of deep facial defects with a single layer dermal regeneration template. J Eur Acad Dermatol Venereol.

[48] Neligan PC. (2013). Head and neck reconstruction. Plast Reconstr Surg.

[49] Felcht M., Koenen W., Sassmann G., Goerdt S., Faulhaber J. (2011). Two-stage reconstruction of head and neck defects after tumor resection with a dermal regeneration template. J Cutan Med Surg.

[50] Hoinoiu T., Grujic D., Prilipceanu G., Folescu R., Hoinoiu B., Bratu T. (2020). The use of collagen-glycosaminoglycan biodegradable matrix (Integra®) in the management of neck postburn hypertrophic scars and contractures. Appl Sci.

[51] Gaviria J.L., Gómez-Ortega V. (2018). One-stage reconstruction of neck burns with single-layer dermal matrix. Plast Aesthet Res.

[52] Mujahid A.M., Tarar F.A., Khalid F.A., Sajjad Y., Ishaque U., Tarar MN. (2021). Outcome of successful graft take of Integra and split thickness skin graft after the release of post-burn neck contracture. Prof Med J.

[53] Hunt J.A., Moisidis E., Haertsch P. (2000). Initial experience of Integra in the treatment of post-burn anterior cervical neck contracture. Br J Plast Surg.

[54] Smith R.M., Byrne PJ. (2019). Reconstruction of the ear. Facial Plast Surg Clin North Am.

[55] Berghaus A., Nicoló MS. (2015). Milestones in the history of ear reconstruction. Facial Plast Surg.

[56] Reiffel A.J., Sohn A.M., Henderson P.W., Fullerton N., Spector JA. (2012). Use of Integra and interval brachytherapy in a 2-stage auricular reconstruction after excision of a recurrent keloid. J Craniofac Surg.

[57] Daya M., Anderson I., Troyer M., Portnof J. (2018). Two step reconstruction of traumatic ear skin avulsion using Integra graft. J Stomatol oral Maxillofac Surg.

[58] Ghazi B.H., Williams JK. (2011). Use of Integra in complex pediatric wounds. Ann Plast Surg.

[59] Plana N.M., Kalmar C.L., Cheung L., Swanson J.W., Taylor JA. (2022). Pediatric dog bite injuries: a 5-year nationwide study and implications of the COVID-19 pandemic. J Craniofac Surg.

[60] May J.M., Depani M., Ferry A.M., Koshy J.C., Thornton JF. (2022). The use of biologic wound agents in pediatric reconstructions. Semin Plast Surg.

[61] Silberstein E., Pagkalos V.A., Landau D., Berezovsky A.B., Krieger Y., Shoham Y. (2014). Aplasia cutis congenita: clinical management and a new classification system. Plast Reconstr Surg.

[62] Aldekhayel S.A., Sinno H., Gilardino MS. (2012). Acellular dermal matrix in cleft palate repair: an evidence-based review. Plast Reconstr Surg.

[63] Kumbla P.A., Yuen J.C., Tait MA. (2015). Applying a dermal regenerative template in management of congenital melanocytic nevi of the hand. Plast Reconstr Surg Glob Open.

[64] Greenhalgh D.G., Hinchcliff K., Sen S., Palmieri TL. (2013). A ten-year experience with pediatric face grafts. J Burn Care Res.

[65] Konofaos P., Kashyap A., Wallace RD. (2014). Total scalp reconstruction following a dog bite in a pediatric patient. J Craniofac Surg.

[66] Groos N., Guillot M., Zilliox R., Braye FM. (2005). Use of an artificial dermis (Integra) for the reconstruction of extensive burn scars in children. About 22 grafts. Eur J Pediatr Surg.

[67] Stiefel D., Schiestl C., Meuli M. (2010). Integra artificial skin for burn scar revision in adolescents and children. Burns.

[68] Weathers W.M., Bhadkamkar M., Wolfswinkel E.M., Thornton JF. (2013). Full-thickness skin grafting in nasal reconstruction. Semin Plast Surg.

[69] Berezovsky A.B., Pagkalos V.A., Silberstein E., Shoham Y., Rosenberg L., Krieger Y. (2015). Primary contraction of skin grafts: a porcine preliminary study. Plast Aesthetic Res.

[70] Mogedas-Vegara A., Agut-Busquet E., Yébenes Marsal M., Luelmo Aguilar J., Escuder de la Torre Ò (2021). Integra as firstline treatment for scalp reconstruction in elderly patients. J Oral Maxillofac Surg.

[71] Khan M.A.A., Ali S.N., Farid M., Pancholi M., Rayatt S., Yap LH. (2010). Use of dermal regeneration template (Integra) for reconstruction of full-thickness complex oncologic scalp defects. J Craniofac Surg.

[72] Corradino B., Di Lorenzo S., Leto Barone A.A., Maresi E., Moschella F. (2010). Reconstruction of full thickness scalp defects after tumour excision in elderly patients: our experience with Integra dermal regeneration template. J Plast Reconstr Aesthet Surg.

[73] Bernstein J.L., Premaratne I.D., Levy A.S., Kuhel W.I., Kutler D.I., Spector JA. (2020). Reconstruction of full thickness scalp defects in extremely elderly patients using dermal regeneration templates. J Craniofac Surg.

[74] Schiavon M., Francescon M., Drigo D., Salloum G., Baraziol R., Tesei J. (2016). The use of integra dermal regeneration template versus flaps for reconstruction of full-thickness scalp defects involving the calvaria: a cost-benefit analysis. Aesthetic Plast Surg.

[75] Voigt D.W., Paul C.N., Edwards P., Metz P. (2006). Original research economic study of collagen-glycosaminoglycan biodegradable matrix for chronic wounds. Wounds.

[76] Jacques E., Suuronen E.J. (2020). The progression of regenerative medicine and its impact on therapy translation. Clin Transl Sci.

[77] Moiemen N.S., Staiano J.J., Ojeh N.O., Thway Y., Frame JD. (2001). Reconstructive surgery with a dermal regeneration template: clinical and histologic study. Plast Reconstr Surg.

[78] Brown B.C., McKenna S.P., Siddhi K., McGrouther D.A., Bayat A. (2008). The hidden cost of skin scars: quality of life after skin scarring. J Plast Reconstr Aesthet Surg.

[79] Moiemen N., Yarrow J., Hodgson E., Constantinides J., Chipp E., Oakley H. (2011). Long-term clinical and histological analysis of Integra dermal regeneration template. Plast Reconstr Surg.

[80] Reynolds M., Kelly D.A., Walker N.J., Crantford C., Defranzo AJ. (2018). Use of Integra in the management of complex hand wounds from cancer resection and nonburn trauma. Hand (N Y).

